# Performance of large language models at the MRCS Part A: a tool for medical education?

**DOI:** 10.1308/rcsann.2023.0085

**Published:** 2023-12-01

**Authors:** A Yiu, K Lam

**Affiliations:** ^1^ King’s College Hospital NHS Foundation Trust, UK; ^2^ Imperial College London, UK

**Keywords:** MRCS, Examination, Large language model, ChatGPT, Bard, Education

## Abstract

**Introduction:**

The Intercollegiate Membership of the Royal College of Surgeons examination (MRCS) Part A assesses generic surgical sciences and applied knowledge using 300 multiple-choice Single Best Answer items. Large Language Models (LLMs) are trained on vast amounts of text to generate natural language outputs, and applications in healthcare and medical education are rising.

**Methods:**

Two LLMs, ChatGPT (OpenAI) and Bard (Google AI), were tested using 300 questions from a popular MRCS Part A question bank without/with need for justification (NJ/J). LLM outputs were scored according to accuracy, concordance and insight.

**Results:**

ChatGPT achieved 85.7%/84.3% accuracy for NJ/J encodings. Bard achieved 64%/64.3% accuracy for NJ/J encodings. ChatGPT and Bard displayed high levels of concordance for NJ (95.3%; 81.7%) and J (93.7%; 79.7%) encodings, respectively. ChatGPT and Bard provided an insightful statement in >98% and >86% outputs, respectively.

**Discussion:**

This study demonstrates that ChatGPT achieves passing-level accuracy at MRCS Part A, and both LLMs achieve high concordance and provide insightful responses to test questions. Instances of clinically inappropriate or inaccurate decision-making, incomplete appreciation of nuanced clinical scenarios and utilisation of out-of-date guidance was, however, noted. LLMs are accessible and time-efficient tools, access vast clinical knowledge, and may reduce the emphasis on factual recall in medical education and assessment.

**Conclusion:**

ChatGPT achieves passing-level accuracy for MRCS Part A with concordant and insightful outputs. Future applications of LLMs in healthcare must be cautious of hallucinations and incorrect reasoning but have the potential to develop AI-supported clinicians.

## Introduction

The Intercollegiate Membership of the Royal College of Surgeons examination (MRCS) is a high-stakes ‘gatekeeper’ for entry to higher surgical specialty training in the UK.^1^ It comprises two parts: Part A, a written component; and Part B, an objective structured clinical examination.^2^ MRCS Part A assesses generic surgical sciences and applied knowledge using 300 multiple-choice Single Best Answer (SBA) items, which are answered in a five-hour examination across two papers on the same day.^3^ It was sat by 5,531 candidates in the 2021/2022 academic year,^4^ and has been noted to exert personal, social and financial costs on trainees in addition to its implications for training progression.^5,6^ There is, therefore, significant interest among surgical trainees in tools that may improve performance at MRCS Part A.

Large Language Models (LLMs) have recently attracted considerable attention owing to their ability to understand, generate and summarise a wide range of natural language inputs at scale.^7,8^ LLMs are deep learning models that are trained to process and understand language patterns using vast amounts of text data, using supervised and reinforcement machine learning techniques. This is used to generate natural language outputs by predicting the likelihood of the next word in a sequence based on the context provided by the preceding words.^9^

Applications of LLMs in healthcare are not, at present, widespread, owing to concerns over data privacy, interpretability, and safety and ethical considerations. As LLMs are limited by their training inputs, there are concerns that the generated outputs may be convincingly incorrect, ‘hallucinated’, or otherwise clinically or socially inappropriate.^10–13^ Current translational work using LLMs has focused primarily on their summary capabilities; for example, generating imaging pathway decisions based on clinical presentation inputs and generating discharge summaries for patients moving from secondary to primary care.^14,15^ LLMs have also shown the ability to generate accurate responses to complex inputs, as demonstrated by impressive performance in a number of professional examinations, including the MBA degree examination,^16^ the United States Certified Public Accountant examination,^17^ the Uniform Bar examination,^18^ and, in a healthcare setting, the United States Medical Licensing Examination (USMLE).^19^

The rapidly increasing competency of LLMs must, therefore, be urgently responded to with clear definitions of their roles in education and assessment. This is especially pressing with the integration of LLM tools into standard services, such as web browsers, productivity software and email clients, further increasing their accessibility to learners.^20^ Rather than taking steps to prevent or obstruct the use of LLMs in education and assessment, many higher education institutions are working to embrace these tools, given the inevitability of their use. This, however, raises important pedagogical, ethical, operational and financial issues, which must be addressed by these institutions.^21^ Especially in healthcare education, where the aim is to produce safe and competent healthcare professionals, use of LLMs must focus on applications that facilitate and/or accelerate learning rather than as shortcuts to passing assessments.

In this study, we evaluate the performance of two publicly available LLMs, ChatGPT (Open AI, San Francisco, CA, USA) built upon GPT-4 (trained upon data up to September 2021) and Bard (Google AI, San Francisco, CA, USA) built upon PaLM2, on their performance on questions from the MRCS Part A. MRCS Part A is suitable for LLM testing as the question items require interpretation of multimodal clinical data and selection between multiple closely related options, and the examination undergoes regular standardisation and has demonstrated stable summary descriptive statistics over a number of years.^3,22^ We aim to assess the accuracy of LLMs in a mock MRCS Part A examination, and consider the concordance and insight displayed by the outputs to determine their value as an educational tool.

## Methods

The methodology chosen was adapted from one previously published by Kung *et al*.^19^

### Question sources

Questions used for the MRCS Part A examination are not publicly available. Therefore, a mock paper consisting of 300 questions deemed representative of the examination was taken from a popular question bank^23^ used widely by surgical trainees preparing for the examination, suggesting reasonable fidelity. All questions were screened to ensure they did not contain visual information, such as clinical imaging or graphs, and were also checked through a Google search to ensure data were not available as training data for either LLM prior to input. Following this screening process, all 300 questions proceeded to be encoded for input into the LLM pipeline.

### Question encoding

Questions were encoded into two formats for input. Firstly, multiple-choice questions were copied verbatim, and the LLM was asked to select the most appropriate option without need for justification (NJ). Secondly, multiple-choice questions were inputted with an additional prompt asking the LLM to provide justification why the option selected was correct and why the other options selected were wrong (J). A new chat session was initiated in the LLM for each entry in order to reduce memory retention bias.

### Pass mark

We used a pass mark range taken from the September 2022, January 2023 and May 2023 MRCS Part A examinations.^22^

### Assessment

LLM outputs were independently scored according to three criteria: accuracy; concordance; and insight, by two surgical trainees who had previously passed the MRCS Part A examination. Responses were classed as accurate if the option chosen was correct, inaccurate if the option chosen was incorrect and indeterminate if the LLM selected more than one option. Responses were classed as concordant for NJ questions if the explanation corresponded with the option chosen and for J questions if the explanation corresponded with the option chosen and negated other options. Finally, the responses were assessed for the number of insights contained. Insights were classified as nondefinitional (where a term was not simply defined in the input question), unique (where a point was made to remove several options), nonobvious (where knowledge or deduction separate to the question was needed) and valid (clinically or numerically accurate points).

Assessors were blinded to each other’s responses. To ensure inter-rater reliability (IRR), 10% of questions included in both Paper 1 (*n*=18) and 2 (*n*=12) were double marked, totalling 120 responses across all four streams. Disagreement was resolved by consensus discussion, and IRR was calculated through Cohen’s kappa statistic.

### Statistics

Basic descriptive statistics were performed, and between-group differences were tested using Fisher’s exact, chi-squared and *t*-tests. All statistics and chart generation were performed using R and ggplot2 through RStudio version 3.6.3 (R Studio, Boston, MA, USA).

## Results

### ChatGPT achieves passing-level accuracy at MRCS Part A (Figure 1)

ChatGPT achieved passing-level accuracy for both NJ and J encodings, achieving 85.7% and 84.3%, respectively (Table 1). Bard achieved 64% for NJ encodings and 64.3% for J encodings. ChatGPT outperforms Bard at both NJ (*p*<0.001) and J (*p*<0.001) encodings. Pass marks for sittings in the period between September 2021 and January 2023 have ranged between 67% and 73%, and therefore ChatGPT achieves passing level accuracy whereas Bard does not. The majority of LLMs scored higher on Paper 1 compared to Paper 2, though this did not reach statistical significance (*p*=0.137). Higher accuracy levels were also achieved across the majority of individual topics in Paper 1 when compared with Paper 2. Inter-rater reliability as calculated by Cohen’s kappa was high at 0.927.

**Figure 1:**
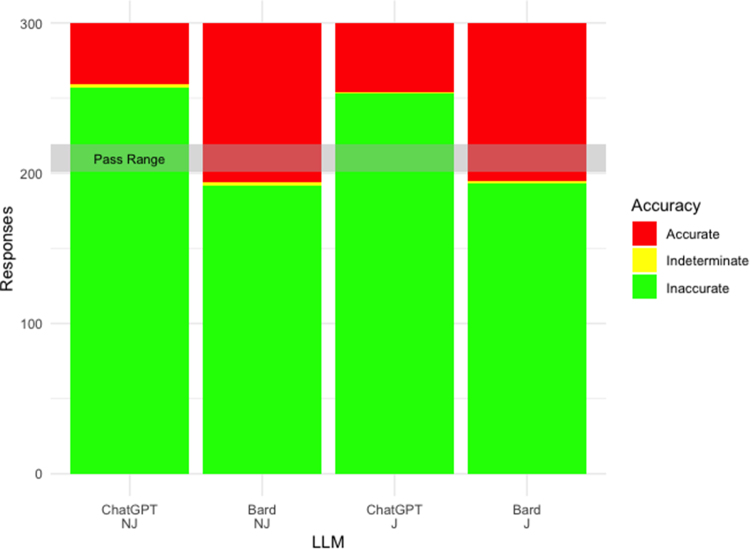
Proportion of accurate responses by ChatGPT and Bard at Membership of the Royal College of Surgeons (MRCS) Part A

**Table 1:** Breakdown of accurate responses by ChatGPT and Bard at Membership of the Royal College of Surgeons (MRCS) Part A by category and encoding

Category	ChatGPT-NJ	ChatGPT-J	Bard-NJ	Bard-J
Applied surgical anatomy	69/82 (84.1%)	66/82 (80.5%)	54/82 (65.9%)	54/82 (65.9%)
Pathology and microbiology	50/55 (90.9%)	50/55 (90.9%)	40/55 (72.7%)	39/55 (70.9%)
Physiology and pharmacology	35/41 (85.4%)	34/41 (82.9%)	27/41 (65.9%)	24/41 (58.5%)
Miscellaneous	2/2 (100%)	1/2 (50%)	1/2 (50%)	2/2 (100%)
Paper 1 total	156/180 (86.7%)	151/180 (83.9%)	122/180 (67.8%)	119/180 (66.1%)
Common surgical conditions	48/50 (96%)	46/50 (92%)	32/50 (64%)	33/50 (66%)
Perioperative management	29/36 (80.6%)	30/36 (83.3%)	19/36 (52.8%)	20/46 (43.5%)
Trauma	24/32 (75%)	26/32 (81.3%)	19/32 (59.4%)	21/32 (65.6%)
Miscellaneous	0/2 (0%)	0/2 (0%)	0/2 (0%)	0/2 (0%)
Paper 2 total	101/120 (84.2%)	102/120 (85%)	70/120 (58.3%)	74/120 (61.7%)
Total	257/300 (85.7%)	253/300 (84.3%)	192/300 (64%)	193/300 (64.3%)

Topic of questions match the themes of the questions found in the examination.

### LLMs achieve high concordance (Figure 2)

Responses to NJ encodings achieved higher concordance compared to J encodings. NJ encodings for ChatGPT were concordant for 286 of 300 (95.3%), while those for Bard were concordant for 245 of 300 (81.7%) (*p*<0.001). J encodings for ChatGPT were concordant for 281 of 300 (93.7%), while those for Bard were concordant for 239 of 300 (79.7%) (*p*<0.001). Both ChatGPT (99.8%) and Bard (98.7%) achieved near perfect concordance rates (*p*=0.09) when producing accurate responses, while concordance rates for inaccurate responses were 66.7% and 49.2% for ChatGPT and Bard, respectively (*p*=0.007). Both LLMs therefore demonstrate high levels of concordance between answer and explanation and therefore high internal consistency.

**Figure 2:**
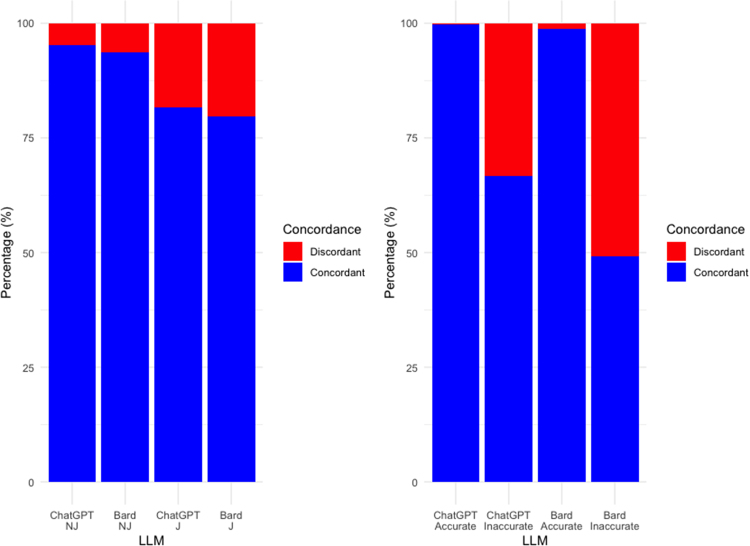
Concordance levels of ChatGPT and Bard: left - breakdown by encoding; right - breakdown by accuracy.

### LLMs provide insightful responses (Figure 3)

Responses were deemed to contain insights if statements were deemed nondefinitional, nonobvious, unique or valid. ChatGPT provided an insightful statement in over 98% of responses, while Bard achieved this for over 86% of responses (*p*<0.001). There was minimal difference between the proportion of responses containing one or more insights between NJ and J encodings (*p*=0.826). However, in order to quantify the number of insights contained in each response, the density of insights metric (quantified by the number of insights divided by the number of responses+1) was analysed for each LLM and encoding. Accurate responses were found to have a greater number of insights than inaccurate responses across all encodings 0.778 vs 0.323 (*p*<0.001). J responses contained a higher number of insights compared with NJ responses for both ChatGPT-J and ChatGPT-NJ (mean=0.866, 0.564, *p*<0.001) and Bard-J and Bard-NJ (mean=0.682, 0.605, *p*=0.009). This demonstrates the ability of LLM to potentially generate text of educational value.

**Figure 3:**
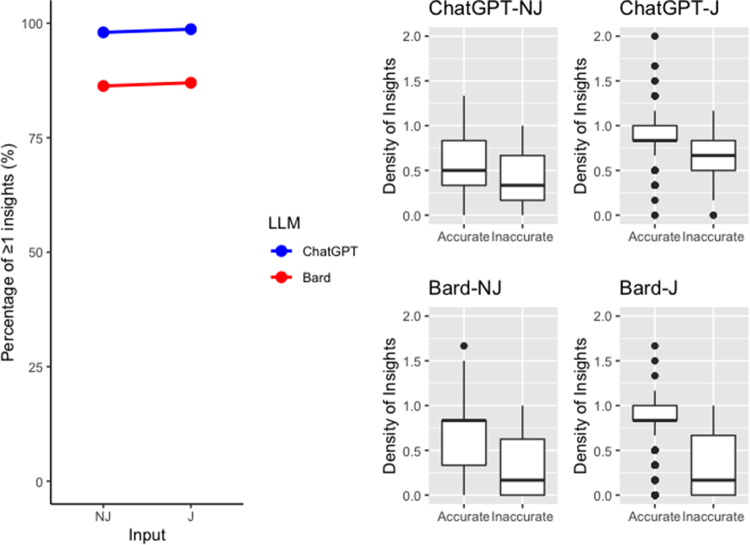
Insight levels of ChatGPT and Bard: left - proportion of insightful responses as defined by number of insights ≥1; right - box plot showing the relationship between density of insights and accuracy across LLM encodings.

### LLMs can display imperfect reasoning

Some responses displayed inconsistent, flawed or incorrect reasoning. Relevant examples are displayed below.

When asked about antibiotic choice for a patient with an erythematous and discharging abdominal split-thickness skin graft (STSG), responses from both LLMs discussed the relative antimicrobial spectra of the antibiotic options given as rational for their choices. The ChatGPT-NJ and -J responses accurately selected the correct option with concordant and insightful outputs; however the Bard-NJ and -J responses were inaccurate. The Bard-NJ response displayed accurate antimicrobial spectral definitions but flawed clinical reasoning, with the J response exposing financial considerations being given a clinically inappropriate level of importance:

*…Ceftriaxone is a third-generation cephalosporin antibiotic that is effective against a wide range of bacteria, including some anaerobic bacteria. However, it is more expensive than metronidazole and is not always necessary for infection after an STSG.*

*Meropenem is a carbapenem antibiotic that is effective against a wide range of bacteria, including some anaerobic bacteria. However, it is the most expensive option and is only used in cases where other antibiotics have failed or are not suitable…*

When asked for the next management step for a patient in class III hypovolaemic shock despite 2.5l of fluid resuscitation, responses from both LLMs highlighted the importance of achieving vital organ perfusion. The ChatGPT responses accurately selected noradrenaline as the most appropriate intervention having acknowledged that the patient had already received adequate fluid resuscitation, while Bard responses selected further fluid resuscitation, with the explanation provided by the Bard-J response showing overall understanding of the management pathway but failure to correctly identify the relevant step:

*Adrenaline (option A) and noradrenaline (option E) are both vasopressors that can be used to raise blood pressure. However, they are not the first-line agents in septic shock… the most appropriate next step in the management of this patient is to give her further intravenous fluids… If her blood pressure does not improve after receiving fluids, then vasopressors may be needed.*

When asked about the initial management of a patient with symptomatic hypercalcaemia with ECG changes, ChatGPT accurately identified intravenous normal saline as the first-line treatment to be commenced for the patient and commented that bisphosphonates are not typically first-line, whereas Bard selected intravenous zolendronic acid and refuted a role for saline administration:

*…normal saline solution… is used to replace fluids and electrolytes. It will not lower the calcium level.*

When asked to interpret an arterial blood gas showing a partially compensated respiratory acidosis, the Bard-J response included:

*…An HCO_3_ of 32mEq/l is within the normal range of 22–26mEq/l…*

There was also one Bard-NJ response to a question regarding acid–base abnormality following excessive resuscitation with normal saline in which the model appeared to incorrectly use the term ‘alkalosis’ in place of ‘acidosis’:

*A hyperchloraemic metabolic **alkalosis** is characterized by an increase in the plasma chloride concentration and a decrease in the plasma bicarbonate concentration. This results in a decrease in the blood pH, which is a measure of acidity…*

All responses to a question regarding the first management step for an intubated patient with decreasing oxygen saturations and absent breath sounds in a hemithorax during a log roll examination inaccurately related to treatment of a suspected pneumothorax, rather than the correct option to adjust the endotracheal tube.

All responses to a question asking about a patient with a suspected pneumothorax causing haemodynamic compromise incorrectly identified needle thoracostomy in the 2nd intercostal space (ICS) as the initial management, rather than the correct option of needle thoracostomy in the 5th ICS.

## Discussion

In this study, we have demonstrated that ChatGPT achieves passing-level accuracy at MRCS Part A and that ChatGPT and Bard achieve high concordance and provide insightful responses to questions requiring interpretation of complex multimodal clinical data.

This is the first application of LLMs to the MRCS Part A Examination, to our knowledge. ChatGPT, using the GPT-4 model, impressively achieved 85.7% accuracy with unprompted inputs. This was achieved without domain-specific training and with minimisation of memory retention bias. This score is comfortably higher than recent pass marks and suggests this LLM’s accuracy in handling complex medical and clinical information continues to rise with model iteration.^19^ Bard, using the PaLM2 model, achieved 64% accuracy with unprompted inputs. Although not reaching the lower bound of the passing range, this result remains remarkable and progression towards or even exceeding the passing range can reasonably be expected with continued maturation of the model given the trajectory of other LLMs.

Interestingly, when asked to justify its responses, ChatGPT’s accuracy reduced to 84.3% while Bard’s accuracy increased to 64.3%. The decrease in ChatGPT’s accuracy replicates findings by Kung *et al* for USMLE Step 2CK, although in their study ChatGPT’s accuracy increased between NJ and J inputs for USMLE Steps 1 and 3.^19^ Understanding why these discrepancies occur between NJ and J encodings will be important to ensure reliability prior to wider uptake of these LLMs.

ChatGPT-NJ and Bard-NJ and -J performance in Paper 1, testing anatomy, physiology, pathology, pharmacology and microbiology, was slightly better than in Paper 2, testing common surgical conditions, trauma and perioperative management. This is possibly due to greater emphasis on factual recall in Paper 1, whereas questions in Paper 2 have a greater focus on knowledge applications such as diagnosis and management planning, which are a greater challenge for LLMs. It is of interest, however, that regardless of question type, LLMs were able to recall relevant knowledge in response.

Forced justification for J encodings provided important insights into LLM reasoning in clinical settings. As foundation LLMs trained on general content, ChatGPT and Bard reflect generic rather than specialist approaches to inputs. This resulted in responses that did not weigh decision-making factors in a clinically appropriate manner, as highlighted by the aforementioned responses to the question of antibiotic choice for a patient with a likely STSG infection. There was some evidence of the LLM failing to appreciate more nuanced clinical scenarios, as demonstrated by the responses to the question on the management of a patient in refractory hypovolaemic shock. There were also instances where responses displayed clinically inaccurate justifications, as in the response to the question on a patient with hypercalcaemia. We also noted discordance in some responses related to clinical chemistry interpretation.

We speculate that the difficulties faced by LLMs in responding to questions regarding specific management steps, such as the question on an intubated patient who desaturates on log rolling, reflects a potential bias in the generic training datasets to emphasise content on pathological diagnoses rather than on-the-job practicalities. We are also concerned that LLMs may not distinguish between current and outdated guidance in their training datasets, as suggested by their failure to appreciate the change in Advanced Trauma Life Support^®^ (ATLS^®^) recommendation for chest decompression from 2nd ICS in the 9th edition to 5th ICS in the 10th edition in response to the question on the initial management of a patient with haemodynamic compromise due to a pneumothorax.^24^

Given the performance of these LLMs in the MRCS Part A Examination, investigation of LLM applications in surgical, and broader medical, education is warranted. Education is an ideal sphere in which to develop LLM utilisation in healthcare as there is a relatively lower potential for harm compared with clinical healthcare settings. Publicly available LLMs such as ChatGPT and Bard are easily accessible and thus provide a time-efficient tool to generate summaries and explanations of relevant topics and practice questions. Bard is freely available while ChatGPT operates a ‘freemium’ model in which access to the GPT-3.5 engine is free but access to the GPT-4 engine requires a $20USD/month subscription.^7,8,25^ However, current LLM builds have the capacity to generate hallucinations, in which incorrect information is presented as true. This was demonstrated in our study and has been widely discussed elsewhere.^26^ It is important for future LLM iterations to further develop basic insights in order to be able to appreciate these errors and for the development of training algorithms to ensure outputs follow the latest clinical guidance. It is also essential for users to maintain appropriate caution and independent oversight when utilising these tools. Eventually, there may be a role for LLMs in the generation of question items with surrounding explanations and educational content.

Perhaps most strikingly, the advent of artificial intelligence (AI) with high-level capabilities in handling complex clinical data to the extent that it is able to pass postgraduate professional examinations, such as MRCS Part A, could herald a paradigm shift in the concepts of medical education and assessment in preparation for clinical practice where factual recall is less emphasised and trustworthy smart decision support tools are commonly available. A good example of an assessment that has successfully integrated use of tools reflective of current clinical practice is the Prescribing Safety Assessment, where candidates have access to digital and hard copies of the British National Formulary, which they can “consult… for relevant information that might be considered beyond… core knowledge”.^27^ Such modern approaches in medical examination are particularly timely with the planned UK-wide introduction of a new Medical Licensing Assessment by the General Medical Council in the 2024–2025 academic year.^28^ To better understand the role of LLMs in academia, the Association for Computational Linguistics recently announced a policy on AI writing assistance, which aims to encourage transparency in LLM usage;^29^ adoption of similar policies among higher education institutions would benefit learners, teachers and assessors as use of LLMs in education continues to grow.

Nevertheless, a number of issues with LLM use must also be considered. Over-reliance on LLMs to supplement clinical reasoning and critical thinking would have significant pedagogical implications on the healthcare workforce and may result in healthcare professionals taking on ethical and legal responsibility for LLM outputs based on training datasets and algorithms that they do not fully understand. Users must also appreciate that information they input into LLMs will be used by companies in accordance with their privacy policy, and as such confidentiality cannot be guaranteed; this is an especially important consideration for the use of LLMs in direct patient-care contexts. Furthermore, LLMs may not remain freely accessible, so practices dependent on LLM usage could potentially incur significant costs in the future. There should also be concern over the environmental impact of LLM usage, with the carbon footprint of ChatGPT recently estimated to be 23kgCO_2_e per day.^30^

Our study has a number of limitations. Stratification of questions according to a cognitive hierarchy, such as Bloom’s taxonomy,^31^ may allow a deeper analysis of the level of thinking demonstrated by the LLMs. Although no questions were excluded in our study owing to the required input of clinical imaging or graphs, the lack of data evaluating performance of LLMs where visual information is inputted represents a potential limitation. Encouragingly, the recent release of GPT-4V allowing multimodal data input including clinical imaging may allow wider use of this technology.^32^ Use of failure modes and effects analysis to evaluate LLM outputs may further explain the causes for inaccurate and/or discordant answers.^33^ Although our output assessment was blinded and demonstrated high IRR, this process is error-prone and at risk of variability and bias. Further work assessing the utility of LLMs as medical education tools in real-world settings is required to evaluate application in these contexts, including generalisability, cost-effectiveness and resource demand.

## Conclusion

Our study is the first application of LLMs to the MRCS Part A Examination, to our knowledge. We have demonstrated that ChatGPT is able to generate accurate, concordant and insightful responses to questions requiring interpretation of complex multimodal clinical data to a degree that achieves passing-level accuracy for the examination without any training or reinforcement. Current LLMs, nevertheless, demonstrate instances of hallucinations, incongruent clinical reasoning and practice in accordance with outdated guidance. Future applications of LLMs in medical education as accessible and efficient tools to summarise and explain the vast body of clinical knowledge may instigate a paradigm shift in medical assessment towards the production of AI-supported clinicians.

## Data availability

The datasets generated during the current study are available from the corresponding author on reasonable request.

## Ethics

Due to the nature of this study, ethical approval was not required.

## Author contributions

AY and KL contributed to the concept and design of the study, acquisition, analysis and interpretation of data, and drafted the manuscript.

## Funding

KL is supported by a NIHR Academic Clinical Fellowship.
